# Supplementary material for: Infants and Newborns with Atypical Teratoid Rhabdoid Tumors (ATRT) and Extracranial Malignant Rhabdoid Tumors (eMRT) in the EU-RHAB Registry: A Unique and Challenging Population
Source: Cancers (Basel). 2022 Apr 27;14(9):2185. doi: 10.3390/cancers14092185 (PMC9100752; doi:10.3390/cancers14092185)
Supplement: Supplementary file 1 [file cancers-14-02185-s001.zip › cancers-1663352-supplementary.pdf]

Supplemental Figure S1

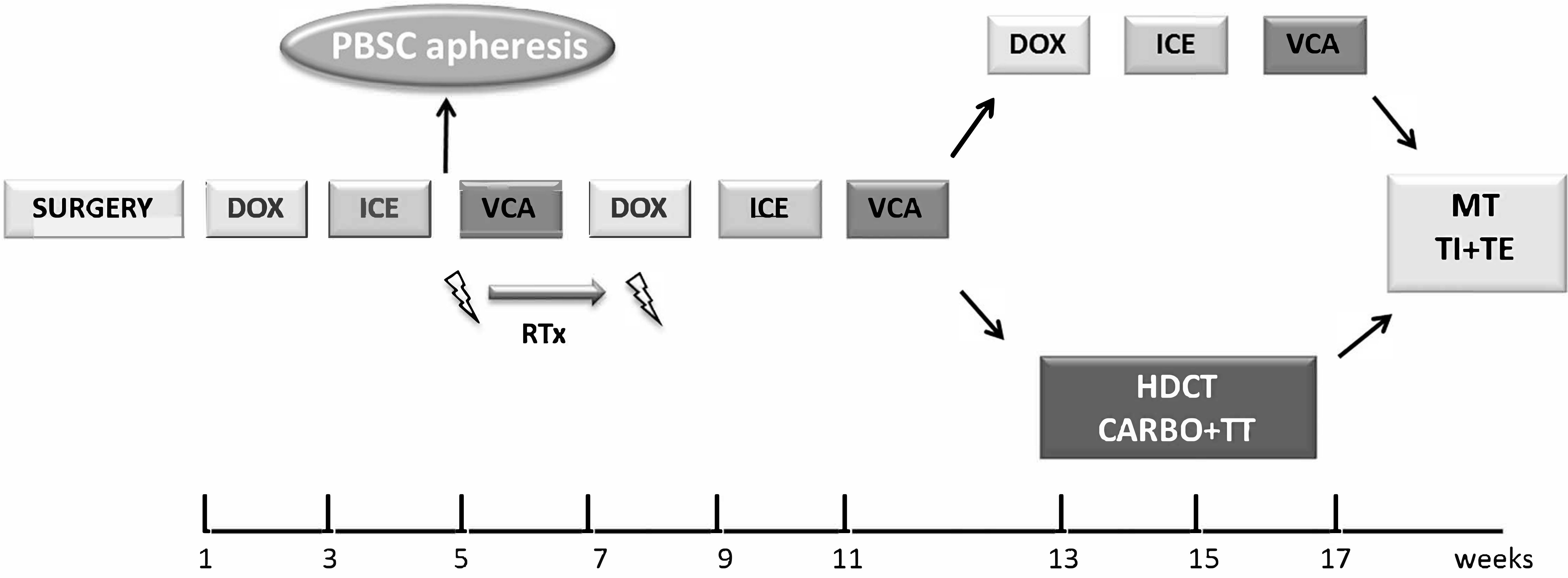

Supplemental Table S1. Conventional chemotherapy courses and high-dose chemotherapy according EU-RHAB

**Conventional Chemotherapy**				
DOX (Doxorubicin)				
Day	Doxorubicin	Methotrexate Intraventricular Therapy (MTX IT)*		
1	37.5 mg/m²	MTX		
2	37.5 mg/m²	MTX		
3		MTX		
4		MTX		
ICE (Ifosfamide, Carboplatinum, Etoposide)				
Day	Ifosfamide	Carboplatinum	Etoposide	MTX IT*
1	2000 mg/m² over 1 h	500 mg/m² over 1 h	100 mg/m² over 1 h	MTX
2	2000 mg/m² over 1 h		100 mg/m² over 1 h	MTX
3	2000 mg/m² over 1 h		100 mg/m² over 1 h	MTX
4				MTX
VCA (Vincristine, Cyclophosphamide, Actinomycin-D)				
Day	Vincristine	Cyclophosphamide	Actinomycin-D	MTX IT*
1	1.5 mg/m² max 2 mg	1500 mg/m² over 1 h	25 µg/kg	MTX
2			25 µg/kg	MTX
3				MTX
8	1.5 mg/m² max 2 mg			
**High-Dose Chemotherapy (HDCT)**				
Day	carboplatinum	thiotepa	Peripheral blood stem cell	
-6	500 mg/m²/d	300 mg/m² 1 h		
-5	500 mg/m²/d	300 mg/m² 1 h		
-4	500 mg/m²/d	300 mg/m² 1 h		
-3				
0			X	

*MTX Age-dependent dose (applied via rickham reservoir):

Dose in mg	< 2 year	2-3 years	> 3 years
MTX	0.5 mg	1 mg	2 mg
